# Biosynthesis of Silver Nanoparticles Produced Using *Geobacillus* spp. Bacteria

**DOI:** 10.3390/nano13040702

**Published:** 2023-02-11

**Authors:** Kotryna Cekuolyte, Renata Gudiukaite, Vaidas Klimkevicius, Veronika Mazrimaite, Andrius Maneikis, Egle Lastauskiene

**Affiliations:** 1 Institute of Biosciences, Life Sciences Center, Vilnius University, Sauletekis Avenue 7, LT-10257 Vilnius, Lithuania; 2 Institute of Chemistry, Faculty of Chemistry and Geosciences, Vilnius University, Naugarduko 24, LT-03225 Vilnius, Lithuania; 3 Faculty of Electronics, Vilnius Gediminas Technical University, Sauletekis Avenue 11, LT-10223 Vilnius, Lithuania

**Keywords:** AgNPs, *Geobacillus* bacteria, nitrate reductase

## Abstract

Silver nanoparticles (AgNPs) are well known for their unique physical and chemical properties, which can be incorporated into a wide range of applications. The growing resistance of microorganisms to antimicrobial compounds promoted the use of AgNPs in antimicrobial therapy. AgNPs can be obtained using physical and chemical methods, but these technologies are highly unfriendly to nature and produce large amounts of side compounds (for example, sodium borohydride and *N,N*-dimethylformamide). Therefore, alternative technologies are required for obtaining AgNPs. This report focuses on the biosynthesis of silver nanoparticles through the reduction of Ag^+^ with the cell-free secretomes of four *Geobacillus* bacterial strains, namely, 18, 25, 95, and 612. Only a few studies that involved *Geobacillus* bacteria in the synthesis of metal nanoparticles, including AgNPs, have been reported to date. The silver nanoparticles synthesized through bio-based methods were characterized using UV–Vis spectroscopy, scanning electron microscopy (SEM), dynamic light scattering (DLS), and zeta potential measurements. UV–Vis spectroscopy showed a characteristic absorbance peak at 410–425 nm, indicative of AgNPs. SEM analysis confirmed that most nanoparticles were spherical. DLS analysis showed that the sizes of the obtained AgNPs were widely distributed, with the majority less than 100 nm in diameter, while the zeta potential values ranged from −25.7 to −31.3 mV and depended on the *Geobacillus* spp. strain.

## 1. Introduction

Different physical, biological, and chemical methods have been used for the synthesis of metal nanoparticles, including silver nanoparticles. Physical and chemical methods are typically expensive and harmful to the environment because they produce toxic waste [1]. Biological methods of nanoparticle synthesis, involving microorganisms and plants, are beneficial because they use non-toxic and environmentally friendly substrates and have a relatively easier synthesis process [2]. Microorganisms are capable of capturing target ions from their environment and turning them into elemental metals through enzymatic reactions [3]. Additionally, biomolecules act as natural stabilizers for nanoparticles, resulting in reduced aggregation and improved stability of NPs during the synthesis process if compared with typically applied chemical methods [4,5].

New methods using microorganisms in the biosynthesis of nanoparticles are expected to play a key role in many conventional and emerging technologies [6]. Many studies have shown that microorganisms can produce various inorganic nanoparticles, including silver nanoparticles (AgNPs), through either intracellular or extracellular processes [7,8,9,10]. Extracellular synthesis of AgNPs by microorganisms has more advantages because it is easier to control the synthesis environment. Additionally, such a type of synthesis requires fewer steps if compared with the intracellular synthesis method [11].

Bacteria of the genus *Geobacillus* are of significant industrial interest owing to their potential use in biotechnological processes as a source of various thermostable enzymes [12]. There are several reports that suggest the use of *Geobacillus* bacteria for the synthesis of metal nanoparticles, including AgNPs [13,14,15,16]; however, the choice of strains is still very limited. It is also suggested that one of the enzymes responsible for the synthesis of AgNPs is nitrate reductase. However, there are not many data on the nitrate reductases of bacteria of the genus *Geobacillus*. At this moment, only sequences of these enzymes and their genes can be obtained in gene/protein databases, but nitrate reductase enzymes are of increasing interest to researchers owing to their thermostability, which allows them to remain active at high temperatures [17]. In addition, *Geobacillus* bacteria are nonpathogenic microorganisms and grow in thermophilic conditions, which lowers the risk of microbial contamination and reduces the steps of AgNPs’ synthesis, as it is not necessary to purify the bacterial enzymes [15,18]. These properties make AgNPs obtained using bacteria of the genus *Geobacillus* potentially useful in both industry and medicine. Thus, this study seeks to expand the theoretical knowledge as well as the practical availability of the use of *Geobacillus* bacteria for AgNP synthesis, as well as to present the biosynthesis of AgNPs using four new *Geobacillus* strains. The activity of nitrate reductases in the selected *Geobacillus* strains and the influence of NADH on the synthesis of AgNPs are discussed in this work.

## 2. Materials and Methods

### 2.1. Source of Microorganisms

The *Geobacillus* strains used in the biogenic synthesis of AgNPs were strains 18, 25, 95, and 612. These strains were isolated in the Department of Microbiology and Biotechnology, Institute of Biosciences (former Faculty of Natural Sciences), Life Sciences Center, Vilnius University (Lithuania), from an oil well. The strain *Geobacillus* sp. 95 was deposited in the DSMZ culture collection under the number DSM 104629 [19]. For the primary screening, *Geobacillus* spp. strains 19, 76, and 481 were also tested. They were isolated and saved in the Department of Microbiology and Biotechnology.

### 2.2. Extracellular Synthesis of Silver Nanoparticles

The synthesis methodology of AgNPs was prepared according to [20,21,22]. The tested *Geobacillus* strains were grown aerobically in 100 mL liquid broth with the following composition (in g/L): tryptone (Roth, Karlsruhe, Germany), 10; meat extract (Merck, Rahway, NJ, USA), 5; NaCl (Merck), 5; CaCl_2_ (Merck), 2.3 mM; and ZnSO_4_ (Merck), 0.91 µM, in 250 mL Erlenmeyer flasks. The cultures were grown in an orbital shaker (Esco, Singapore, Singapore) at 55 °C with aeration at 180 rpm. After 48 h of incubation, cells were separated by centrifugation at 16,000× *g* for 10 min. Cell-free secretomes were used as material for AgNP synthesis. The secretomes of the target strains were treated with AgNO_3_ (Roth) solution at final concentrations of 2 mM. The whole mixtures were incubated in a shaking incubator for 48 h at 55 °C and 200 rpm. The secretomes without AgNO_3_ and bacterial growth medium supplemented with 2 mM AgNO_3_ were used as controls. After 48 h of incubation, the mixtures were centrifuged at 3000× *g* for 10 min to remove media components. Then, mixtures were centrifuged at 16,000× *g* for 15 min to collect AgNPs. To remove unconverted silver ions, the obtained pellets were washed three times with 70% ethanol and three times with deionized water by centrifugation at 16,000× *g* for 15 min. 

### 2.3. Nitrate Reductase Assay

After cells of *Geobacillus* spp. strains 18, 25, 95, and 612 were separated by centrifugation at 16,000× *g* for 10 min, the resulting secretomes were used for the nitrate reductase assay. The nitrate reductase assay was adapted based on Harley [23]. An amount of 2 mL of a secretome was distributed to each test tube. The activity was measured at temperatures ranging from 5 to 90 °C. Nine control samples were prepared by adding 2 mL of assay medium (30 mM KNO_3_ (Roth) and 5% propanol (Roth) in 0.1 M phosphate buffer pH 7.5) to the secretome and then immediately placing the samples into boiling water for 5 min to inhibit the enzymatic activity. After cooling the samples and adding 2 mL of *N*-(1-naphthyl) ethylenediamine dihydrochloride (NEED, 0.02% (w/v) (Roth)) in distilled water) and 2 mL of sulfanilamide (SA, 1% (w/v) (Roth)) in 25% (v/v) HCl (Roth)), the control tubes were kept at room temperature until the nitrite concentration was measured. After adding the assay medium to the tested secretomes, they were incubated in the dark for 60 min at the tested temperature, before placing the tubes in boiling water for 5 min. After cooling the samples, 2 mL of SA and 2 mL of NEED were added. Then, samples were incubated for 20 min at room temperature and the absorbance at 540 nm was measured. The enzymatic activity was expressed as μmol of nitrite * h^−1^ * L^−1^. The nitrite standard curve was produced by placing different known amounts of potassium nitrite (from 0 to 250 nmol NO_2_) followed by 5 mL of NEED and SA solutions. 

### 2.4. Characterization of Silver Nanoparticles

The formation of the AgNPs was monitored using UV–Vis spectral analysis. The spectra of cell-free secretomes treated with 2 mM AgNO_3_ were measured at 300–600 nm using an ultraviolet–visible (UV–Vis) spectrophotometer (Ultrospec 5300 pro, Amersham Biosciences Corp., Piscataway, NJ, USA). The size, distribution, and morphology of the nanoparticles were further analyzed via scanning electron microscopy (SEM) (Tescan Vega 3, Brno - Kohoutovice, Czech Republic). Samples for SEM were prepared by drop-coating the AgNP solution onto an aluminum grid. Before transferring samples to the microscope, they were dried at 55 °C. The zeta potential values of AgNPs were measured using a Malvern Zetasizer Nano ZS (Malvern Panalytical, Malvern, UK) and recalculated applying the Smoluchowski model. The AgNP particle size distribution was evaluated using the dynamic light scattering (DLS) method (ZetaSizer Nano ZS (Malvern Panalytical, Malvern, UK) equipped with a 4 mW He-Ne laser emitting at a wavelength of 633 nm). Measurements were performed at 25 °C and an angle of 173°. These parameters were determined using AgNPs at a concentration of 1 mg/mL in water.

### 2.5. NADH Assay

In order to determine whether NADH is sufficient for the synthesis of AgNPs, a mixture of Tris-HCl (pH 8) buffer (AppliChem, Darmstadt, Germany), 2 mM AgNO_3_, and 0.2 mM NADH (Roth) was prepared. The reaction was carried out for 48 h, after which UV–Vis analysis was performed in the same manner as described in Section 2.4.

## 3. Results

### 3.1. Extracellular Biosynthesis and Characterization of Obtained Silver Nanoparticles

**Figure 1:**
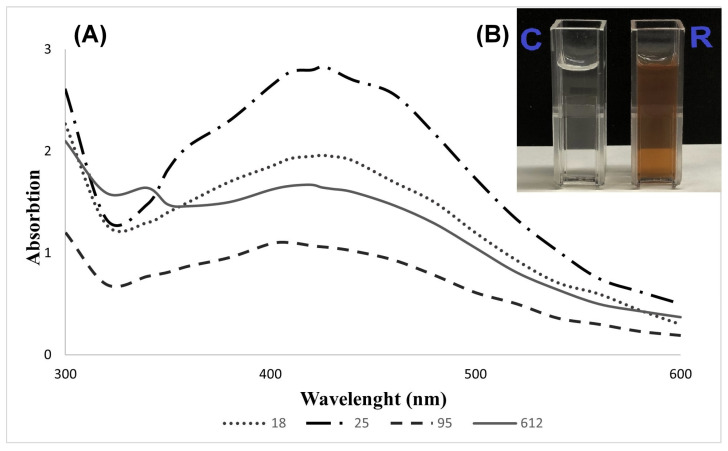
UV–visible spectrum analysis of AgNPs originating from secretomes of *Geobacillus* spp. 18, 25, 95, and 612 treated with 2 mM AgNO_3_ after 48 h incubation at 55 °C (**A**). 18—*Geobacillus* sp. 18; 25—*Geobacillus* sp. 25; 95—*Geobacillus* sp. 95; 612—*Geobacillus* sp. 612. Color change of a reaction using the *Geobacillus* sp. 612 secretome (**B**). R—reaction mix; C—control.

In this study, four *Geobacillus* spp. strains (18, 25, 95, and 612) were chosen for further biosynthesis of silver nanoparticles (AgNPs). In the primary screening using a nitrate reductase assay and secretomes of *Geobacillus* spp. bacteria, seven strains (18, 19, 25, 76, 95, 481, and 612) were tested; however, strains 19, 76, and 418 did not show nitrate reductase activity (results not presented). Further experimental data showed that the extracellular synthesis of AgNPs was successfully induced in the secretomes of *Geobacillus* strains 18, 25, 95, and 612. After 48 h of treatment with 2 mM aqueous AgNO_3_ solution at 55 °C, the color shift from light brown to dark brown in the secretomes was detected. This change indicated the reduction of Ag^+^ ions and the formation of AgNPs. The synthesized AgNPs were analyzed using UV–Vis spectroscopy. UV–Vis spectroscopy is a widely used technique for the structural characterization of AgNPs [14]. After 48 h of exposure to 2 mM AgNO_3_, the samples containing *Geobacillus* spp. cell-free secretomes were analyzed using UV–Vis at 300–600 nm (Figure 1). All four tested samples showed a characteristic AgNP absorbance peak at 410–425 nm. A color shift from light brown to greenish brown was detected in the bacterial growth medium (control sample) supplemented with 2 mM AgNO_3_; however, no precipitation of aggregates was detected after centrifugation.

**Figure 2:**
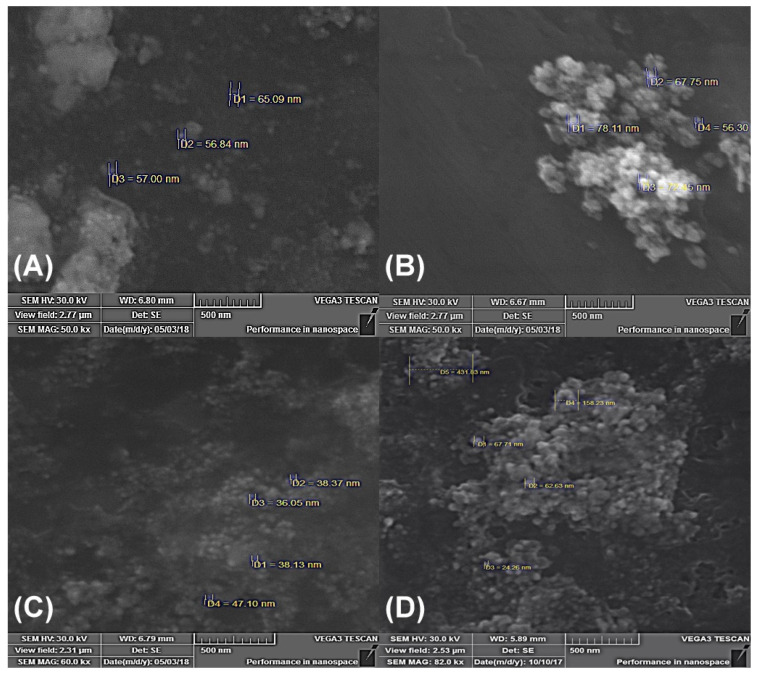
SEM analysis of obtained AgNPs. (**A**) *Geobacillus* sp. 18; (**B**) *Geobacillus* sp. 25; (**C**) *Geobacillus* sp. 95; (**D**) *Geobacillus* sp. 612.

Further SEM analysis (Figure 2) of the AgNPs revealed that the synthesized particles were spherical. It was also noticed that, during the drying process of the AgNPs, agglomeration occurred, leading to the formation of distinct nanostructures (Figure 2).

The size and the size distribution of the obtained AgNPs were evaluated using DLS analysis. A total of 100 separate measurements were performed for AgNPs obtained using different *Geobacillus* spp. strains. The results show that the diameter of the majority of the particles in the aqueous dispersion was <100 nm (82% of *Geobacillus* sp. strain 18; 99% of strain 25; 75% of strain 95; 88% of strain 612). However, the particle size distribution was relatively wide (please refer to Figure S1 in ESI), and some aggregates were detected. The results of the distribution sizes allow to assume that the larger particles obtained are most likely aggregates of smaller AgNPs (this is also shown by the SEM analysis in Figure 2). To explain the particle aggregation in the dispersions, additional zeta potential measurements were performed at 25 °C applying the Smoluchowski model. The results are summarized in Table 1.

The results show that the zeta potentials of each AgNP obtained using the four *Geobacillus* spp. strains were negative and varied between −25 and −31 mV (Table 1). It is well established that NPs in aqueous dispersions are stable if the absolute zeta potential value exceeds 30 mV (ξ > +30 mV or ξ < −30 mV) [24]. The zeta potential results correspond well with the theory because the AgNPs obtained using the *Geobacillus* spp. strains with the highest negative zeta potential value of −31.3 ± 0.8 mV had the lowest number of aggregates (ca. 1% of all particles) in the aqueous dispersion. For particles obtained using other *Geobacillus* spp. strains, the surface charge was less expressed, and electrostatic repulsion forces between particles were insufficient to ensure efficient stability. In such cases, a higher number of aggregated particles in the dispersions was detected during the DLS measurements. For more information, please refer to Figure S1 in ESI.

### 3.2. Nitrate Reductase Activity

**Figure 3:**
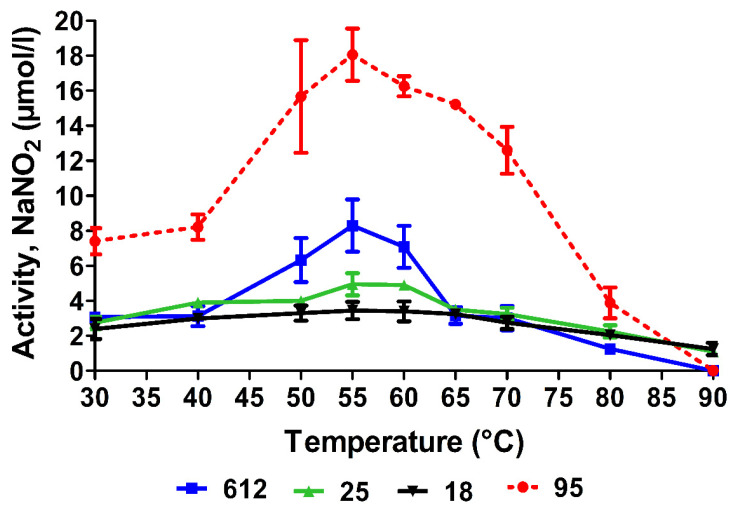
Nitrate reductase activity from different *Geobacillus* spp. strains at various temperatures. The activity is presented as the amount of formed NaNO_2_ (μmol/L). 18, 25, 95, and 612—strain numbers.

In the further experiments, the effect of temperature on the activity of nitrate reductases from all four strains was investigated in more detail (Figure 3). The highest nitrate reductase activity was observed in *Geobacillus* sp. 95. The highest nitrate reductase activity was detected at 55 °C, while significantly high activity was detected in the temperature range of 50–70 °C. The nitrate reductase activity in the other three strains, 18, 25, and 612, was approximately 2–4 times lower. The obtained data confirmed that the optimal temperature for *Geobacillus* spp. bacterial nitrate reductases is in the range of 50–60 °C, and the enzyme of strain 95 is characterized by higher activity (Figure 3). In addition, a correlation was observed between the nitrate reductase activities of the different *Geobacillus* spp. strains and the amount of AgNPs formed. Using *Geobacillus* sp. strain 95 with the highest nitrate reductase activity yielded about three times more AgNPs than using *Geobacillus* spp. strains 18 and 25 with the lowest enzyme activity.

**Figure 4:**
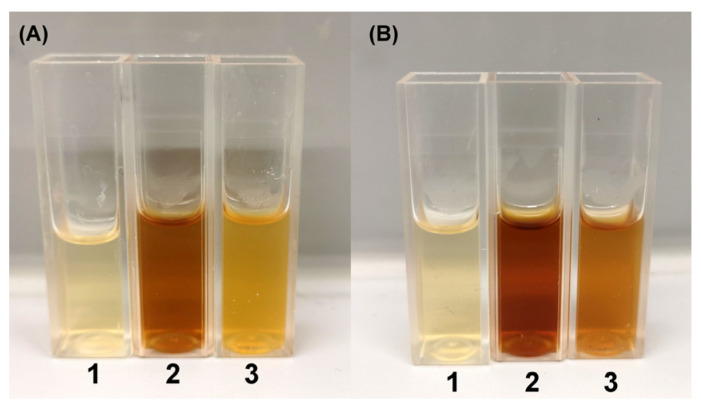
The evaluation of AgNP formation using *Geobacillus* spp. bacterial secretomes after 24 h (**A**) and 48 h (**B**). 1—bacterial growth medium without *Geobacillus* spp. cells supplemented with 2 mM of AgNO_3_; 2—strain 95 secretome supplemented with 2 mM of AgNO_3_; 3—strain 612 secretome supplemented with 2 mM of AgNO_3_.

Based on the results that the nitrate reductase of *Geobacillus* sp. strain 95 has the highest activity, it was hypothesized that the formation time of AgNPs would be shorter using the secretome of *Geobacillus* sp. 95 compared with that of the other strains (Figure 4). For further experiments, *Geobacillus* sp. strain 612 was chosen as a strain with low nitrate reductase activity. A visible color change with the secretome of *Geobacillus* sp. strain 95 was observed after incubation for 24 h (Figure 4A), while with that of *Geobacillus* sp. 612, this was observed after 48 h (Figure 4B).

**Figure 5:**
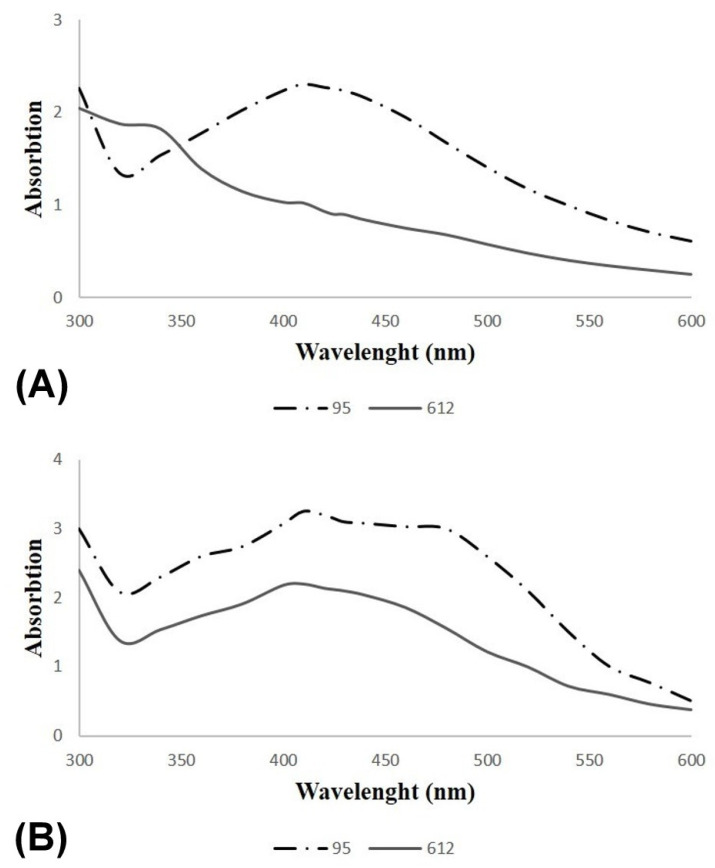
UV–visible spectrum analysis of AgNPs originating from secretomes of *Geobacillus* spp. 95 and 612 treated with 2 mM AgNO_3_ after 24 h incubation (**A**) and 48 h incubation (**B**) at 55 °C.

UV–Vis analysis was also performed to evaluate the AgNPs obtained using *Geobacillus* spp. strains 95 and 612 after 24 h and 48 h (Figure 5). The obtained results confirm the results of the determination of the color change, as the AgNP-specific peak occurred after 24 h of synthesis with the *Geobacillus* sp. strain 95 secretome (Figure 5A) and after 48 h of synthesis with the *Geobacillus* sp. strain 612 secretome (Figure 5B).

**Figure 6:**
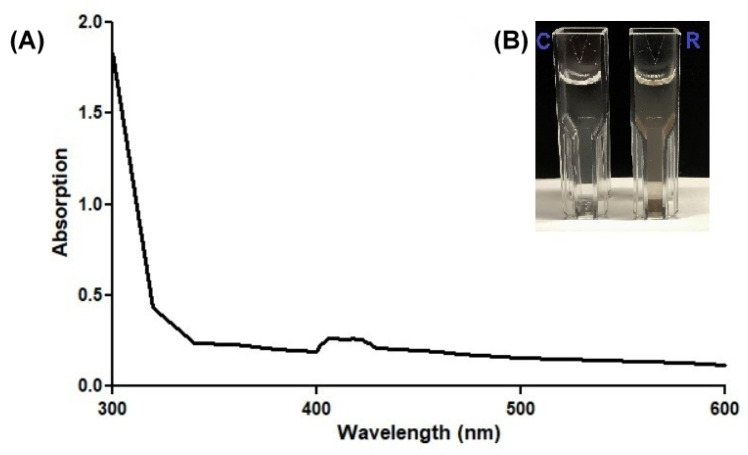
Spectrophotometric analysis of silver reduction without the use of a source of the nitrate reductase enzyme (2 mM AgNO_3_ and 0.2 mM NADH, 48 h incubation at 55 °C temperature) (**A**), and the color change (**B**). R—reaction mix; C—control.

There is evidence in the literature that a mixture of NAD(P)H and AgNO_3_ is sufficient for the synthesis of AgNPs, so this study also evaluated the influence of NADH on the formation of AgNPs without using the source of the nitrate reductase enzyme (*Geobacillus* spp. bacterial secretomes). During the spectrophotometric analysis, a slight particle plasmon resonance (PPR) peak of the NPs was detected (Figure 6). However, the performed experiment shows that NADH is not sufficient for the synthesis of AgNPs.

## 4. Discussion

Silver nanoparticles (AgNPs) are particles that are generally smaller than 100 nm and contain 20–15,000 silver atoms. They have distinct physical, chemical, and biological properties compared with their bulk parent materials. Much research and application attention has been focused on the superior antimicrobial activity of AgNPs, which can help to reduce the use of various antibiotics. Thus, methods for the cheap, simple, and eco-friendly production of AgNPs are very important. Biological methods for synthesizing AgNPs are an alternative to chemical and physical methods. In this paper, the extracellular synthesis of AgNPs using *Geobacillus* spp. secretomes was investigated. This is the first study to comprehensively analyze the potential of bacteria in the genus *Geobacillus* for the synthesis of AgNPs. While previous research had explored the use of these bacteria for AgNP synthesis, this is the first report to thoroughly examine their potential. In this study, four *Geobacillus* spp. strains, namely, 18, 25, 95, and 612, were tested more deeply for AgNP production. All four strains were chosen based on the primary nitrate reductase screening as nitrate reductases are the main enzymes mediating the biosynthesis of AgNPs from AgNO_3_. Moreover, the *Geobacillus* sp. 95 strain has already been successfully explored as a source of highly active, thermostable lipolytic enzymes [19]. Thus, this study highlights the attractiveness of the *Geobacillus* sp. 95 strain as a promising industrial bacterial strain and suggests the possibility of applying it for the biosynthesis of AgNPs.

The primary method used for the detection of AgNPs is the change in the medium color after cell-free secretome incubation with AgNO_3_ [14]. These changes were detected with 2 mM AgNO_3_ in the case of using all four strains of *Geobacillus* spp. cell-free secretomes. The color change from light brown to dark brown is caused by the excitation of surface plasmon vibrations in the AgNPs [25,26,27,28]. The surface plasmon resonance of AgNPs depends on their shape, size, and dielectric environment [29] and, in this study, the resonance peaks were observed in the 410–425 nm range, which was confirmed to be the surface plasmon resonance band of AgNPs [26,30]. A similar absorbance maximum was detected for AgNPs produced using a cell-free extract of *Geobacillus stearothermophilus* [15]. According to Fayaz et al. (2011), the obtained AgNPs were spherical, with a diameter of 5–35 nm, as determined using TEM photographs. The SEM analysis of our study also showed that the form of the obtained AgNPs was spherical, but the particle size distribution was wider (with the majority of AgNPs being <100 nm in size). Moreover, we used DLS analysis, which is a more accurate method for determining the particle size in aqueous dispersions. It is worth noting that the size and morphology of AgNPs can be controlled through various parameters (e.g., temperature) if needed [5]. Further research is needed to optimize the AgNP synthesis conditions so that the sizes of the obtained AgNPs using *Geobacillus* spp. bacteria are not so different. This could be achieved, for example, by determining the time point at which AgNPs are formed using different *Geobacillus* spp. bacterial strains. This is important because, to use these AgNPs in practice, such a wide distribution of particle sizes can lead to non-uniform antimicrobial properties.

It was found that the zeta potentials of the obtained AgNPs were negative, with values ranging from −25 to −31 mV. This is not uncommon, and there are many examples in the literature where negative zeta potential values of AgNPs obtained through biological synthesis have been reported [31,32,33]. The negative zeta potential values may be due to the binding of microorganisms or nutrient media elements, such as proteins and amino acids from the secretome, to the surface of the obtained AgNPs. It is worth noting that the zeta potential is an important parameter that provides information about the stability of the obtained particles, particularly in aqueous dispersions. It has been established that, if the absolute zeta potential value exceeds 30 mV (ξ > +30 mV or ξ < −30 mV), then the formed colloids will be stable in aqueous dispersions, that is, they will not agglomerate [24]. According to the currently available data, the obtained AgNPs are stable for at least 45 days at room temperature. It is worth noting that, because the zeta potential of AgNPs obtained using *Geobacillus* spp. bacteria is around −30 mV, particle aggregation can occur, resulting in a wide distribution of particle sizes. Further studies are needed to determine the stability of the resulting AgNPs over a longer period of time.

Bacteria of other genera can also be used for the synthesis of AgNPs. The most analyzed bacteria belong to the genera *Pseudomonas* and *Bacillus* [34,35]. However, the bacteria of these genera are mesophilic; thus, although this could reduce synthesis costs (i.e., lower temperatures would be used), it also increases the risk that the obtained AgNPs will be contaminated with mesophilic microorganisms, which may produce unwanted products and/or be pathogens. To date, the production of AgNPs using thermophilic *Thermus thermophilus* [36], a *Bacillus* sp. isolated from a hot spring [37], and the thermophilic mold *Sporotrichum thermophile* [38] has been reported. The use of thermophilic bacteria such as *Geobacillus* for AgNP synthesis can help to protect the AgNP synthesis process from contamination and to decrease the amount of other microbiological by-products. Therefore, AgNPs synthesized using thermophilic bacteria could be applied not only in industry, but also in medicine.

AgNPs have a broad application range. They are used for the production of sterilizing material in consumable and medical products, including textiles, food storage bags, refrigerator surfaces, and personal care products. It was estimated that the global market of AgNPs reached a value of USD 2052.10 million in 2021, which is projected to reach USD 6.6 billion by 2030, exhibiting a growth rate (CAGR) of 15.6% from 2021 to 2030. AgNPs can be used in the formulation of surface cleaners, toys, textiles, air and water disinfection, antimicrobial catheters, antimicrobial gels, antimicrobial paints, food packaging supplies, clinical clothing, and so on [39]. AgNP incorporation in nanoscale sensors can offer faster response times and lower detection limits [40]. It has also been proposed to use AgNPs as an adjuvant in the manufacturing of vaccines [41], and it has been shown that AgNPs have both anti-angiogenic and anti-cancer properties [42]. There is a huge market in which AgNPs are needed. Thus, the testing of antimicrobial activity and the introduction of AgNPs produced using the secretomes of *Geobacillus* spp. strains 18, 25, 95, and 612 in sterilizing material or medical products are tasks for further research.

The exact mechanism of AgNP formation using biological systems is still a matter of debate. Some scientific groups claim that nitrate reductase is the main enzyme involved in the reduction of Ag^+^ and the subsequent formation of AgNPs [25,43,44], while others claim that the reduction of Ag^+^ occurs independently of nitrate reductase, involving only NAD(P)H [45,46]. In this study, we demonstrated that NADH alone is not sufficient for the efficient synthesis of AgNPs. Similar results were reported in a study by Li et al. (2012) [3], who found that NADH alone does not produce AgNPs. When nitrate reductase is also involved in the synthesis, the yield of AgNPs is higher than when only NADH is used, which suggests that both components are important for the synthesis of AgNPs using *Geobacillus* spp. bacterial secretomes. In this study, the nitrate reductase activity in *Geobacillus* bacteria was analyzed for the first time, although several genes are available in databases. The majority of research on bacterial nitrate reductase production for use in AgNP biosynthesis focuses on *E. coli* [47,48,49], *Bacillus* [32,43,44,50], and *Pseudomonas* [51,52].

Bacterial nitrate reductases can be classified into three distinct types—periplasmic nitrate reductase (Nap), respiratory nitrate reductase (Nar), and assimilatory nitrate reductase (Nas)—based on their cellular location, operon organization, and active site structure [53]. *Geobacillus* nitrate reductases belong to Nar and are composed of three different subunits, namely, α (narG), β (narH), and γ (narI), having properties of metal-binding regions and domains. The narI gene has been identified to encode the *Geobacillus* nitrate reductase molybdenum cofactor assembly chaperone, suggesting that molybdenum is important for nitrate reductase functionality. To the best of our knowledge, this is all of the available information about *Geobacillus* nitrate reductases. However, for computational profiling experiments of nitrate reductase from *Bacillus clausii*, Mukherjee and co-authors [43] used the catalytic NarG subunit as a representative of the whole nitrate reductase. Thus, it is possible to clone and perform gene and protein engineering experiments of only NarG to improve nitrate reductase activity and increase the AgNP production rate. Both fundamental experiments related to the functionality of nitrate reductase from *Geobacillus* bacteria and the prospect of their application are important objects for further research.

In addition to NP synthesis, other areas of application of nitrate reductases are being investigated. With increasing urbanization and rapid population growth, environmental pollution with heavy metals is a major concern that is directly related to increasing industrialization [54]. Heavy metals are dangerous because they are not biodegradable and are accumulating in the food chain and endangering all living organisms [55]. To remove heavy metals from the environment, chemical precipitation, evaporation, electrochemical treatment, and other methods are used [56], but these methods are not very efficient and consume a lot of chemical compounds and energy [57]. As an alternative, bioremediation methods have been developed, which are more economical and environmentally friendly [58]. One such method is MICP, which involves the precipitation of calcium carbonates by microbial cells and their biochemical activities. MICP occurs under different environmental conditions through various metabolic pathways, including nitrate reduction, which has sparked interest in nitrate reductases [59,60]. As it was demonstrated that *Geobacillus* spp. bacterial secretomes can be used for the synthesis of AgNPs, and their nitrate reductases are active over a wide range of temperatures, further studies are needed to determine whether *Geobacillus* spp. bacterial nitrate reductases could be applied not only to the synthesis of AgNPs, but also to bioremediation.

## 5. Conclusions

This research emphasizes the use of secretomes from *Geobacillus* spp. bacteria as a safe, straightforward, environmentally friendly, and effective method for synthesizing silver nanoparticles. The resulting silver nanoparticles are safe to use and have a range of potential applications, including in the fields of medicine, electronics, and material science.

## Figures and Tables

**Table 1:** Values of the zeta potential (mV) of AgNPs obtained using secretomes of four different *Geobacillus* spp. strains.

Tested Strain of *Geobacillus* spp.	Zeta Potential Values (mV)
18	−26.6 ± 0.5
25	−31.3 ± 0.8
95	−25.7 ± 0.8
612	−27.4 ± 0.6

## Data Availability

Not applicable.

## Supplementary Materials

The following supporting information can be downloaded at https://www.mdpi.com/article/10.3390/nano13040702/s1, Figure S1: Size distribution of AgNPs obtained using four *Geobacillus* spp. bacteria strains. Number refers to the respective *Geobacillus* spp. strain number.
