# Supplementary material for: Biosynthesis of Silver Nanoparticles Produced Using Geobacillus spp. Bacteria
Source: Nanomaterials (Basel). 2023 Feb 11;13(4):702. doi: 10.3390/nano13040702 (PMC9965977; doi:10.3390/nano13040702)
Supplement: Supplementary file 1 [file nanomaterials-13-00702-s001.zip › nanomaterials-2164067-supplementary.pdf]

nanomaterials

Supplementary Materials

Biosynthesis of Silver Nanoparticles Produced Using *Geobacillus* spp. Bacteria

Kotryna Cekuolyte [1], Renata Gudiukaite [1], Vaidas Klimkevicius [2], Veronika Mazrimaite [1], Andrius Maneikis [3], and Egle Lastauskiene [1,*]

[1] Institute of Biosciences, Life Sciences Center, Vilnius University, Sauletekis Avenue 7, LT-10257 Vilnius, Lithuania
[2] Institute of Chemistry, Faculty of Chemistry and Geosciences, Vilnius University, Naugarduko 24, LT-03225 Vilnius, Lithuania
[3] Faculty of Electronics, Vilnius Gediminas Technical University, Sauletekis Avenue 11, LT-10223 Vilnius, Lithuania
* Correspondence: egle.lastauskiene@gf.vu.lt.

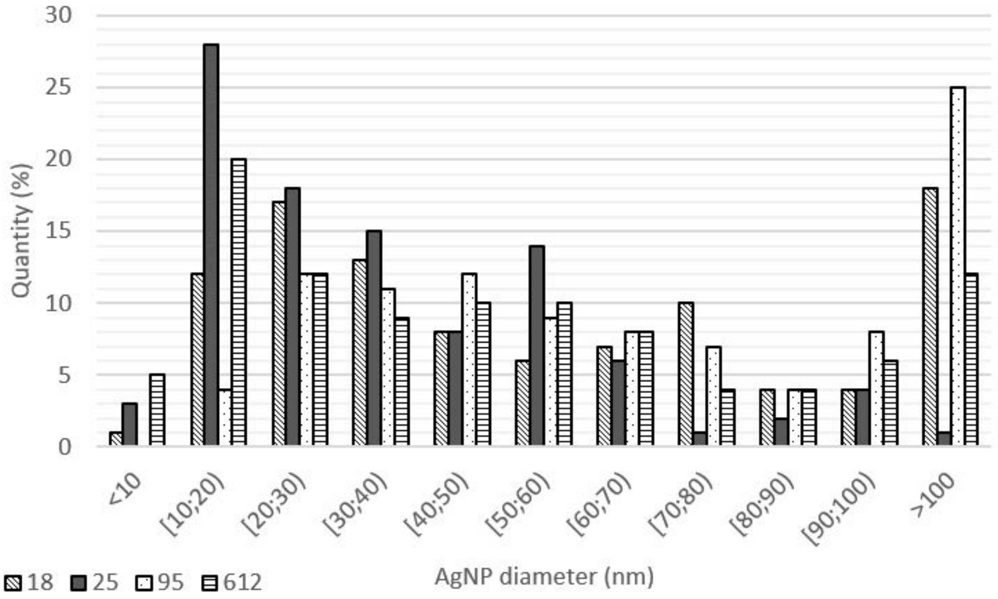

Figure S1. Size distribution of AgNPs obtained using four *Geobacillus* spp. strains. Numbers refer to the respective *Geobacillus* spp. strain numbers.
